# Clinical outcomes of dichorionic diamniotic twin pregnancies following single versus double embryo transfer in human medically assisted reproduction

**DOI:** 10.1093/humrep/deag031

**Published:** 2026-03-05

**Authors:** H Hattori, Y Goto, Y Kasahara, K Kyono

**Affiliations:** Kyono ART Clinic Sendai, Sendai, Miyagi, Japan; Kyono ART Clinic Takanawa, Minato-ku, Tokyo, Japan; Kyono ART Clinic Morioka, Morioka-shi, Japan; HOPE (Human Ovarian-tissue Preservation Enterprise), Shinagawa-ku, Tokyo, Japan; Kyono ART Clinic Sendai, Sendai, Miyagi, Japan; Kyono ART Clinic Sendai, Sendai, Miyagi, Japan; Kyono ART Clinic Takanawa, Minato-ku, Tokyo, Japan; Kyono ART Clinic Morioka, Morioka-shi, Japan; HOPE (Human Ovarian-tissue Preservation Enterprise), Shinagawa-ku, Tokyo, Japan; Kyono ART Clinic Sendai, Sendai, Miyagi, Japan; Kyono ART Clinic Takanawa, Minato-ku, Tokyo, Japan; Kyono ART Clinic Morioka, Morioka-shi, Japan; HOPE (Human Ovarian-tissue Preservation Enterprise), Shinagawa-ku, Tokyo, Japan

**Keywords:** single embryo transfer, double embryo transfer, dichorionic diamniotic twins, monozygotic twins, live birth

## Abstract

**STUDY QUESTION:**

Do clinical and perinatal outcomes of dichorionic diamniotic (DCDA) twin pregnancies differ between single embryo transfer (SET) and double embryo transfer (DET) in human medically assisted reproduction (MAR)?

**SUMMARY ANSWER:**

In DCDA twin pregnancies, SET was associated with a significantly higher incidence of complete miscarriage and a lower rate of twin live births than DET.

**WHAT IS KNOWN ALREADY:**

While DET has historically been the major contributor to dizygotic DCDA twins, the global adoption of SET has markedly reduced such cases. However, monozygotic twinning (MZT) occurs more frequently after MAR, especially in blastocyst transfer cycles, and the prognosis of monozygotic DCDA twins remains poorly understood.

**STUDY DESIGN, SIZE, DURATION:**

This single-center retrospective cohort study analyzed 206 clinical multiple pregnancies achieved between January 2014 and December 2024, following 4658 fresh and 15 872 frozen–warmed embryo transfer cycles.

**PARTICIPANTS/MATERIALS, SETTING, METHODS:**

Only cycles using autologous oocytes were included. Clinical and perinatal outcomes of DCDA twin pregnancies derived from SET and DET were compared. To account for baseline differences between SET and DET groups, an exploratory multivariable logistic regression analysis was performed for clinical outcomes. Statistical analyses were performed using the Mann–Whitney *U*-test and Fisher’s exact test, with *P* < 0.05 considered significant.

**MAIN RESULTS AND THE ROLE OF CHANCE:**

When comparing the clinical course of DCDA twin pregnancies, the incidence of two gestational sacs and two fetal heartbeats was significantly higher in the DET group than in the SET group (98.0% vs 47.2%, *P* < 0.0001; 63.5% vs 25.5%, *P* < 0.0001) (two fetal heartbeats: adjusted odds ratios [aOR], 0.276; 95% CI, 0.108–0.706; *P* < 0.007). Twin live birth occurred in 53.1% of DET-derived DCDA twins and 17.6% of SET-derived DCDA twins (*P* < 0.0001) (aOR, 0.324; 95% CI, 0.121–0.867; *P* = 0.025), whereas complete miscarriage was more frequent after SET (49.0% vs 17.7%, *P* < 0.0001) (aOR, 9.140; 95% CI, 3.030–27.600; *P* < 0.0001). Perinatal outcomes, including gestational age, birth weight, and congenital anomaly rates, did not differ significantly between groups.

**LIMITATIONS, REASONS FOR CAUTION:**

The number of monozygotic cases was limited, and zygosity could not be genetically confirmed. Some same-sex DCDA twins may have been dizygotic in origin.

**WIDER IMPLICATIONS OF THE FINDINGS:**

These findings highlight that DCDA twin pregnancies should not be regarded as a uniform clinical entity in MAR. Even within the same chorionicity category, early outcomes differ significantly between monozygotic twins after SET and dizygotic twins after DET. Although SET remains the optimal strategy to prevent multiple pregnancies, further studies should aim to identify embryos at higher risk of post-transfer splitting and to refine preventive criteria for MZT.

**STUDY FUNDING/COMPETING INTEREST(S):**

There is no funding for this study.

**TRIAL REGISTRATION NUMBER:**

N/A.

## Introduction

In the early years of human medically assisted reproduction (MAR), transferring multiple embryos was a common strategy to maximize pregnancy rates (European IVF Monitoring Consortium for the European Society of Human Reproduction and Embryology; Smeenk *et al.*, 2025). However, this led to a sharp rise in multiple pregnancies, particularly twin gestations. Numerous studies have consistently demonstrated that twin pregnancies carry a substantially higher risk of severe perinatal complications for both the mother (e.g. hypertensive disorders of pregnancy, gestational diabetes, postpartum hemorrhage) and the offspring (e.g. preterm birth, low birth weight, perinatal mortality, cerebral palsy) compared with singleton pregnancies (Obiechina *et al.*, 2011; Eapen *et al.*, 2020; Alteri *et al.*, 2024). To mitigate these serious risks, single embryo transfer (SET) has become the standard strategy worldwide (European IVF Monitoring Consortium for the European Society of Human Reproduction and Embryology; Smeenk *et al.*, 2025). This paradigm shift has led to a marked reduction in the incidence of dizygotic dichorionic diamniotic (DCDA) twin pregnancies, which primarily resulted from double embryo transfer (DET). While the decline in DET has successfully reduced the occurrence of dizygotic DCDA twins, accumulating evidence indicates that monozygotic twinning (MZT) occurs more frequently following MAR, particularly in blastocyst transfer cycles, than in natural conception, with reported incidences ranging from 0.97 to 2.35% (Vitthala *et al.*, 2009; Busnelli *et al.*, 2019).

Perinatal outcomes of twin pregnancies are largely determined by chorionicity and amnionicity. DCDA twins generally carry a lower risk of miscarriage and perinatal complications than monochorionic twins (Feng *et al.*, 2018; Litwinska *et al.*, 2020). In contrast, monochorionic diamniotic (MCDA) twins are at risk of complications such as twin–twin transfusion syndrome, while monochorionic monoamniotic (MCMA) twins are associated with cord entanglement and a markedly higher perinatal risk (D’Antonio *et al.*, 2019; Litwinska *et al.*, 2020; Rissanen *et al.*, 2022). Among MZT cases, previous studies have reported that MCDA twins occur most frequently, or that MCDA and DCDA twins occur at similar rates, whereas MCMA twins occur less often (Kyono, 2013; Otsuki *et al.*, 2016). Recent studies have challenged the classical embryological theory that chorionicity is determined within the first 3 days post-fertilization. Instead, evidence increasingly supports a mechanism in which embryonic splitting occurs at the blastocyst stage, involving either the inner cell mass (ICM) or the entire embryo (Kyono, 2013; Sundaram *et al.*, 2018; Jin *et al.*, 2024). Furthermore, MAR-related procedures, such as zona pellucida (ZP) manipulation, vitrification, blastocyst transfer, and blastocyst grading, have been implicated as potential contributors to MZT formation (Alikani *et al.*, 1994; Shibuya and Kyono, 2012; Hviid *et al.*, 2018; Ikemoto *et al.*, 2018; Hattori *et al.*, 2019; Chen *et al.*, 2023). Consequently, the increasing incidence of MZT following SET remains a major clinical concern.

Importantly, DCDA twins originating from SET and DET may arise from distinct origins and carry different risk profiles depending on the MAR treatment performed. DCDA twins following DET are predominantly dizygotic, resulting from the implantation of two separately transferred embryos. In contrast, the vast majority of DCDA twins following SET are monozygotic, arising from the splitting of a single embryo, with dizygotic DCDA twins after SET occurring only in extremely rare cases due to coincidental spontaneous conception (Kyono *et al.*, 2009; Osianlis *et al.*, 2014; Jin *et al.*, 2024). Although monozygotic DCDA twinning after SET is thought to result from post-blastocyst division, a mechanism fundamentally different from that of dizygotic DCDA twinning, evidence regarding their clinical outcomes, such as miscarriage and live birth rates, remains limited.

Despite sharing the same DCDA chorionicity, twin pregnancies following SET and DET are presumed to differ substantially in zygosity, which may translate into distinct pregnancy and perinatal risk profiles. To clarify the impact of embryo transfer strategies in MAR on clinical outcomes and to inform the management of multiple pregnancies, this single-center cohort study compared pregnancy and perinatal outcomes between DCDA twin pregnancies following SET and those following DET. These were presumed to be predominantly monozygotic and dizygotic, respectively.

## Materials and methods

### Study design and patients

This retrospective cohort study included 206 cases of clinical multiple pregnancies achieved between January 2014 and December 2024 at a single infertility treatment center, following 4658 fresh embryo transfer cycles and 15 872 frozen–warmed embryo transfer (FET) cycles. In Japan, oocyte donation is still subject to ongoing ethical and societal discussion and is not routinely practiced. As a result, no donor oocyte cycles were included in this cohort, and only cycles using autologous oocytes were analyzed. The study compared the clinical and perinatal outcomes of twin pregnancies resulting from SET and DET cycles. The study protocol was approved by the Institutional Review Board of Kyono ART Clinic on 5 April 2025 (reference number: 6002-250405). All procedures were performed in accordance with the ethical standards of the institutional and national committees on human experimentation and with the 1964 Helsinki Declaration and its later amendments. All participants provided informed consent for the use of anonymized clinical data for research purposes.

### Ovarian stimulation

Controlled ovarian stimulation was performed using recombinant FSH, hMG, or clomiphene citrate. The LH surge was suppressed with either a GnRH agonist or antagonist protocol. Ovulation was triggered with 5000 or 10 000 IU of hCG when the leading follicle reached a mean diameter of 18 mm. Transvaginal oocyte retrieval was performed 36 h after hCG administration under ultrasound guidance.

### ICSI/IVF, embryo culture, and vitrification–warming procedures

ICSI or conventional IVF was performed 2–4 h after oocyte retrieval, depending on semen parameters and previous fertilization outcomes. Embryos were cultured up to Day 6 in Global medium (LifeGlobal, USA) or Continuous Single Culture–NX medium (Fujifilm, Japan). Embryo culture was conducted at 37°C under humidified conditions of 5.0% O_2_, 6.0% CO_2_, and 89.0% N_2_ using either a conventional incubator or a time-lapse culture system. Surplus embryos of good quality, as well as all embryos in freeze-all cycles, were cryopreserved by vitrification using either the Cryotop method (Kitazato, Japan) or the Cryotech method (Reprolife, Japan). Both vitrification and warming procedures were performed according to the manufacturer’s protocol. Blastocysts were morphologically evaluated 1–2 h before transfer using the Istanbul Consensus (Coticchio *et al.*, 2025). High-quality cleavage-stage embryos were defined as those with more than four cells on Day 2 or more than eight cells on Day 3, minimal fragmentation (<10%), and uniform blastomere size. High-quality blastocysts were defined as those with an expansion grade ≥3 and ICM and trophectoderm (TE) grades of A or B. Blastocysts with a TE grade of C were classified as low quality, whereas embryos with ICM grades of C or D and/or a TE grade of D were excluded from embryo transfer.

### Fresh embryo transfer and FET

Embryo transfer was performed at the cleavage stage (Day 2 or 3) or blastocyst stage (Day 5 or 6). For blastocyst transfer, embryo selection was based primarily on the degree of expansion and the quality of the ICM and TE. In FET cycles, either a natural cycle (NC) or a hormone replacement cycle (HRC) was used. In HRC, patients received oral estrogen (Progynova, 6 mg/day; Bayer, Germany) until the endometrium reached a thickness of 8 mm. Subsequently, combined estrogen–progestin therapy was continued using either oral dydrogesterone (30 mg/day; Duphaston, Abbott Japan LLC, Japan) or vaginal progesterone (300 mg/day; Lutinus, Ferring Pharmaceuticals, Switzerland) until a negative pregnancy test or up to 10 weeks of gestation. In NCs, follicular development and hormone levels were monitored until the endometrial thickness reached 8 mm, and the dominant follicle measured 18 mm. Ovulation was induced with hCG, followed by luteal support with progestin. To avoid the occurrence of a concurrent spontaneous pregnancy, patients were instructed to abstain from sexual intercourse during the embryo transfer cycle. Vitrified–warmed embryo transfer was performed on Day 6 after the initiation of progestin supplementation. Assisted hatching (AH) was performed upon patient request for both fresh and frozen–warmed embryos. AH was performed using a laser system, in which a slit was made in the ZP of cleavage-stage embryos, and approximately one-fourth of the ZP was opened in blastocyst-stage embryos. All embryo transfers were performed transcervically under ultrasound guidance using a soft IVF catheter.

### Assessment of multiple pregnancies

In this study, pregnancies achieved through MAR were followed by transvaginal ultrasonography at 1- or 2-week intervals from 5 to 9 weeks of gestation. Chorionicity was initially assessed between 5 and 6 weeks of gestation based on the number of gestational sacs observed on ultrasonography. Amnionicity was evaluated using established ultrasonographic criteria. Pregnancies in which a T-sign was clearly identified were diagnosed as MCDA twins, whereas DCDA twins were identified by the presence of the twin peak sign. Vanishing twin syndrome is defined as the spontaneous reduction of a fetus while still *in utero* (Magnus *et al.*, 2017). In cases where amnionicity could not be determined with confidence, re-evaluation was requested at a tertiary referral center in accordance with the guidelines of the International Society of Ultrasound in Obstetrics and Gynecology (Khalil *et al.*, 2025). In addition, because reporting of pregnancy outcomes following MAR is mandatory in Japan, diagnoses of chorionicity and amnionicity were further verified by cross-checking early ultrasonographic findings with membrane status documented in delivery reports for each case. Among DCDA twin pregnancies derived from SET cycles, cases in which the two fetuses had different sexes were considered dizygotic twins resulting from a concurrent spontaneous conception and were therefore excluded from the analysis. In the present cohort, only one such case was identified and excluded. The remaining SET-derived DCDA twin pregnancies therefore consisted exclusively of same-sex twins.

### Statistical analysis

All statistical analyses were performed using JMP Pro 13.1.0 (SAS Institute, Cary, NC, USA). Continuous variables were compared using the Mann–Whitney *U*-test, and categorical variables were analyzed using Fisher’s exact test. To account for baseline differences between SET and DET groups, an exploratory multivariable logistic regression analysis was performed for clinical outcomes. The model included maternal age, BMI, method of endometrial preparation, embryo stage, use of AH, and embryo quality as covariates. Adjusted odds ratios (aORs) with 95% CIs were calculated. Statistical significance was set at *P* < 0.05.

## Results

### Baseline characteristics

Background data on the subjects in SET cycles and DET cycles are presented in Table 1. During the study period, a total of 19 674 SET cycles and 856 DET cycles were performed. Multiple pregnancies occurred in 108 SET cycles and 98 DET cycles. The mean maternal age was significantly higher in the DET group compared with the SET group (37.7 ± 3.7 vs 36.5 ± 4.0 years, *P* = 0.014). Maternal BMI was also significantly greater in the DET group (21.3 [17.4–31.7]) than in the SET group (20.1 [16.2–27.1], *P* = 0.005). Among FET cycles, natural ovulatory cycles were more frequent in the DET group (45.4%) than in the SET group (12.2%), whereas HRCs were more frequent in the SET group (87.8% vs 54.6%, *P* < 0.0001). At the time of embryo transfer, cleavage-stage transfers were more common in the SET group (10.2%) compared with the DET group (2.0%), while blastocyst transfer predominated in the DET group (98.0%, *P* = 0.004). AH was performed in all DET cycles, significantly more frequently than in the SET group (76.9%, *P* < 0.0001). Regarding embryo morphology, the proportion of high-quality embryos was significantly higher in the SET group (75.0%) than in the DET group (47.4%, *P* < 0.0001), whereas low-quality embryos were more common in the DET group (52.6%). When baseline characteristics were compared specifically between DCDA twin pregnancies derived from SET and DET, similar results were observed.

**Table 1.** Characteristics of study subjects in monozygotic and dizygotic multiple pregnancies.

	All twin pregnancies			DCDA twin pregnancies		
Variable	SET	DET	*P* value	SET	DET	*P* value
No. of multiple pregnancy cycles	108	98	–	51	96	–
Fresh cycles	10	1		6	1	
Frozen cycles	98	97		45	95	
Maternal age (y)	36.5 ± 4.0	37.7 ± 3.7	0.014	35.7 ± 4.5	37.7 ± 3.7	0.008
Paternal age (y)	38.7 ± 5.6	39.7 ± 5.2	0.193	38.2 ± 4.2	39.7 ± 5.1	0.082
Maternal BMI (kg/m^2^)	20.1 ± 3.0	21.3 ± 2.9	0.005	20.2 ± 2.3	21.8 ± 2.9	0.001
Antimüllerian hormone (ng/mL)	2.7 ± 2.0	2.2 ± 1.7	0.072	3.3 ± 2.9	2.2 ± 1.6	0.472
Cause of infertility						
Ovarian reserve factor, n (%)	38 (34.9)	32 (32.7)	0.769	18 (35.3)	30 (31.3)	0.712
Uterine factor, n (%)	8 (7.3)	8 (8.2)	1.000	3 (5.9)	8 (8.3)	0.748
Tubal factor, n (%)	9 (8.3)	14 (14.3)	0.191	5 (9.8)	14 (14.6)	0.606
Male factor, n (%)	39 (35.8)	25 (25.5)	0.131	20 (39.2)	25 (26.0)	0.132
Unexplained, n (%)	15 (13.8)	19 (19.4)	0.459	5 (9.8)	19 (19.8)	0.160
Duration of infertility (y)	2.7 ± 2.4	3.0 ± 2.3	0.197	2.5 ± 2.3	3.1 ± 2.3	0.116
Type of frozen cycles			<0.0001			<0.0001
Natural ovulation cycles	12 (12.2)	44 (45.4)		6 (13.3)	43 (45.3)	
Hormone replacement cycles	86 (87.8)	53 (54.6)		39 (86.7)	52 (54.7)	
Type of insemination (No. of embryos)			0.350			0.428
ICSI	74 (68.5)	145 (74.0)		36 (70.6)	74 (77.1)	
IVF	34 (31.5)	51 (26.0)		15 (29.4)	22 (22.9)	
Type of embryo transfer (No. of embryos)			0.004			0.049
Cleavage-stage	11 (10.2)	4 (2.0)		5 (9.8)	2 (2.1)	
Blastocyst-stage	97 (89.8)	192 (98.0)		46 (90.2)	94 (97.9)	
Assisted hatching			<0.0001			<0.0001
Yes	83 (76.9)	98 (100)		39 (76.5)	96 (100)	
No	25 (23.1)	0		12 (23.5)	0	
PGT			1.000			1.000
Yes	1 (0.9)	0		0	0	
No	107 (99.1)	98 (100)		51 (100)	96 (100)	
Embryo grade (No. of embryos)			<0.0001			<0.0001
High-quality embryos	81 (75.0)	93 (47.4)		39 (76.4)	89 (46.3)	
Low-quality embryos	27 (25.0)	103 (52.6)		12 (23.5)	103 (53.6)	

Data are shown as mean ± SD. DCDA, dichorionic diamniotic; SET, single embryo transfer; DET, double embryo transfer; PGT, preimplantation genetic testing.

### Clinical outcomes according to embryo number in DCDA twin pregnancies

When comparing the clinical course of DCDA twin pregnancies, the incidence of clinical twin gestational sacs was significantly higher in the DET group than in the SET group (98.0% vs 47.2%, *P* < 0.0001; Table 2). Similarly, the proportion of pregnancies in which two fetal heartbeats were confirmed was also higher in the DET group (63.5% vs 25.5%, *P* < 0.0001). The rate of twin live birth, defined as the delivery of two live infants, was significantly greater in the DET group (53.1%) compared with the SET group (17.6%, *P* < 0.0001). Complete miscarriage occurred significantly more frequently in the SET group (49.0%) than in the DET group (17.7%, *P* < 0.0001). Multivariable logistic regression analysis showed that SET-derived DCDA twin pregnancies were significantly associated with lower rates of confirmation of two fetal heartbeats (aOR, 0.276; 95% CI, 0.108–0.706; *P* = 0.007) and twin live birth (aOR, 0.324; 95% CI, 0.121–0.867; *P* = 0.025), as well as a higher risk of complete miscarriage (aOR, 9.140; 95% CI, 3.030–27.600; *P* < 0.0001; Table 3).

**Table 2.** Comparative analysis of clinical outcomes according to chorionicity and amnionicity.

Variable	SET	DET	*P* value
Total multiple pregnancies	108	98	–
DCDA twins			
Two GSs (clinical pregnancy), n (%)	51 (47.2)	96 (98.0)	<0.0001
Two FHBs, n (%)	13 (25.5)	61 (63.5)	<0.0001
Single FHB, n (%)	20 (39.2)	30 (31.3)	0.364
Twin live birth, n (%)	9 (17.6)	51 (53.1)	<0.0001
Single live birth, n (%)	17 (33.3)	28 (29.2)	0.707
Vanishing twin, n (%)	22 (43.1)	37 (38.5)	0.597
Complete miscarriage, n (%)	25 (49.0)	17 (17.7)	<0.0001
MCDA twins			
Two GSs (clinical pregnancy), n (%)	49 (45.4)	0	–
Two FHBs, n (%)	49 (100.0)	–	–
Twin live birth, n (%)	31 (63.3)	–	–
Single live birth, n (%)	4 (8.2)	–	–
Vanishing twin, n (%)	4 (8.2)	–	–
Complete miscarriage, n (%)	14 (28.6)	–	–
MCMA twins			
Two GSs (clinical pregnancy), n (%)	6 (5.6)	0	–
Two FHBs, n (%)	6 (100.0)	–	–
Twin live birth, n (%)	2 (33.3)	–	–
Vanishing twin, n (%)	0	–	–
Complete miscarriage, n (%)	4 (66.7)	–	–
DCDA triplets			
Two GSs (clinical pregnancy), n (%)	1 (0.9)	0	–
Three FHBs, n (%)	1 (100.0)	–	–
Triple live birth, n (%)	1 (100.0)	–	–
Vanishing fetuses, n (%)	0	–	–
Complete miscarriage, n (%)	0	–	–
DCTA triplets			
Three GSs (clinical pregnancy), n (%)	0	2 (2.0)	–
Three FHBs, n (%)	–	2 (100.0)	–
Triple live birth, n (%)	–	1 (50.0)	–
Single live birth, n (%)	–	1 (50.0)	–
Vanishing fetuses, n (%)	–	1 (50.0)^a^	–
Complete miscarriage, n (%)	–	0	–
MCTA triplets			
Single GSs (clinical pregnancy), n (%)	1	0	–
Three FHBs, n (%)	1 (100.0)	–	–
Vanishing fetuses, n (%)	0	–	–
Complete miscarriage, n (%)	1 (100.0)	–	–

SET, single embryo transfer; DET, double embryo transfer; DCDA, dichorionic diamniotic; MCDA, monochorionic diamniotic; MCMA, monochorionic monoamniotic; DCTA, dichorionic triamniotic; MCTA, monochorionic triamniotic; GS, gestational sac; FHB, fetal heartbeat.

a In this case, a set of triplets resulted in a singleton live birth due to the vanishing of two embryos/fetuses.

**Table 3.** Adjusted odds ratios of clinical outcomes in dichorionic diamniotic twin pregnancies.

Clinical outcome	DCDA	AOR	95% CI	*P*-value
Two FHBs	DET	1.000 (reference)		
	SET	0.276	(0.108–0.706)	0.007
Twin live birth	DET	1.000 (reference)		
	SET	0.324	(0.121–0.867)	0.025
Complete miscarriage	DET	1.000 (reference)		
	SET	9.140	(3.030–27.600)	<0.0001

The adjusted variables included mother’s age, BMI, method of endometrial preparation, embryo stage, use of assisted hatching, and embryo quality.

DCDA, dichorionic diamniotic; AOR, adjusted odds ratio; FHB, fetal heartbeat; SET, single embryo transfer; DET, double embryo transfer.

**Figure 1.**
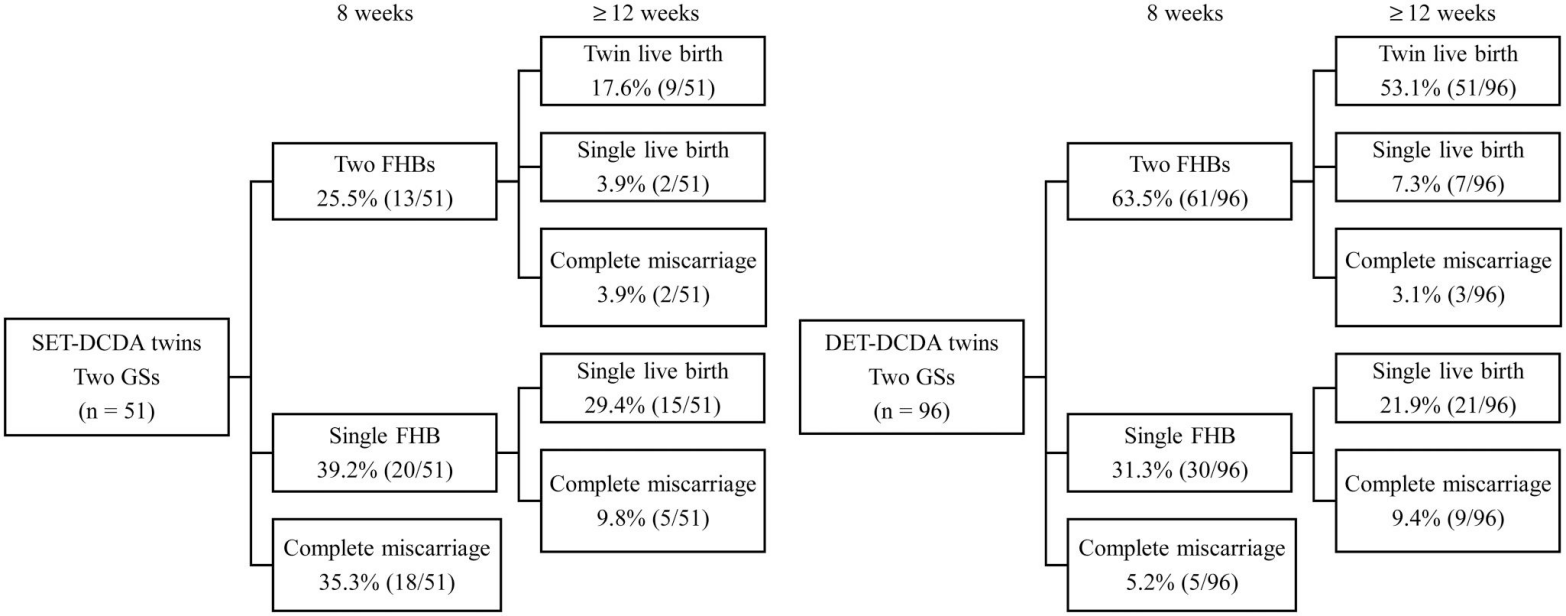
**Detailed clinical outcomes of dichorionic diamniotic twin pregnancies derived from single embryo transfer and double embryo transfer cycles.** The figure illustrates the clinical progression of DCDA twin pregnancies following SET and DET from confirmation of gestational sacs to delivery outcomes. At 8 weeks of gestation, two fetal heartbeats were confirmed in 25.5% of SET-derived DCDA twins and 63.5% of DET-derived DCDA twins. Among DET-derived DCDA twins, 53.1% resulted in twin live births and 17.7% in complete miscarriage, whereas among SET-derived DCDA twins, 17.6% resulted in twin live births and 49.0% in complete miscarriage. SET, single embryo transfer; DET, double embryo transfer; DCDA, dichorionic diamniotic; GS, gestational sac; FHB, fetal heartbeat.

When analyzed by gestational age, among pregnancies in which two fetal heartbeats were confirmed at 8 weeks, 17.6% in the SET group and 53.1% in the DET group resulted in twin live births (Fig. 1). In pregnancies with a single FHB, the single live birth rate was comparable between the groups (29.4% for SET vs 21.9% for DET). Complete miscarriage was markedly more frequent in the SET group (35.3% vs 5.2%), occurring predominantly between 8 and 12 weeks of gestation. Moreover, even after confirmation of two fetal heartbeats, the incidence of complete miscarriage remained higher in the SET group (2/13) than in the DET group (3/61). Conversely, in the DET group, early complete miscarriages were less common, and most pregnancies with confirmed fetal heartbeats continued beyond the first trimester. In summary, DCDA twin pregnancies derived from SET were characterized by significantly poorer early clinical outcomes, including lower rates of confirmation of two fetal heartbeats and twin live birth, and a markedly higher incidence of complete miscarriage, compared with those derived from DET.

### Comparison of clinical outcomes between monozygotic DCDA and MCDA twin pregnancies after SET

In a comparison of outcomes between monozygotic DCDA and MCDA twin pregnancies derived from SET, DCDA twin pregnancies showed a significantly lower rate of confirmation of two fetal heartbeats and twin live birth and a higher rate of complete miscarriage than MCDA twin pregnancies (Table 2). Specifically, the rate of confirmation of two fetal heartbeats was 25.5% in DCDA twins and 100% in MCDA twins (*P* < 0.0001), whereas the complete miscarriage rate was 49.0% and 28.6%, respectively (*P* < 0.0001). Overall, these results demonstrate that, even among monozygotic twin pregnancies conceived through SET, early clinical outcomes differed markedly between DCDA and MCDA twins.

### Perinatal events and congenital anomalies in DCDA twin pregnancies

Perinatal outcomes and neonatal parameters were comparable between the SET- and DET-derived DCDA twin groups (Table 4). There were no statistically significant differences between the two groups in the incidence of perinatal complications, placenta previa, placental abruption, hypertensive disorders of pregnancy, or gestational diabetes mellitus. Gestational age at delivery, birth weight, birth height, and sex ratio were also similar between groups. The rates of low birth weight (<2500 g), preterm birth (<37 weeks), and congenital anomalies did not differ significantly between SET- and DET-derived DCDA twin pregnancies. Among DCDA twin pregnancies that progressed beyond the early gestational period, perinatal outcomes and congenital anomaly rates were comparable between SET- and DET-derived twins.

**Table 4.** Perinatal events and congenital anomalies in dichorionic diamniotic twin pregnancies.

	Singleton			Twin		
	SET	DET	*P* value	SET	DET	*P* value
Infants (n)	17	28	–	18	102	–
Perinatal complications, n (%)	3 (17.6)	5 (17.9)	0.986	4 (22.2)	20 (19.6)	0.798
Placenta previa, n (%)	1 (5.8)	1 (3.6)	0.715	1 (5.6)	2 (2.0)	0.368
Placental abruption, n (%)	0	1 (3.6)	0.431	1 (5.6)	3 (2.9)	0.569
HDP, n (%)	1 (5.9)	2 (7.1)	0.869	1 (5.6)	10 (9.8)	0.565
GDM, n (%)	1 (5.9)	1 (3.6)	0.715	1 (5.6)	5 (4.9)	0.907
Gestational age (week)	38.5 ± 2.6	38.5 ± 2.8	0.062	35.2 ± 2.2	35.3 ± 1.9	0.886
Birth weight (g)	3030 ± 400	3050 ± 400	0.776	2130 ± 520	2242 ± 402	0.359
Birth height (cm)	48.3 ± 2.8	49.8 ± 2.2	0.170	44.2 ± 3.5	44.8 ± 3.0	0.680
Sex ratio (male/female)	2.40 (12/5)	0.87 (13/15)	0.114	0.50 (6/12)	0.92 (49/53)	0.248
Low birth weight, n (%) (<2500 g)	1 (5.9)	1 (3.6)	0.715	12 (66.7)	72 (72.0)	0.738
Preterm birth, n (%) (<37 weeks)	1 (5.9)	1 (3.6)	0.715	8 (44.4)	58 (56.9)	0.329
Congenital abnormalities, n (%)	1 (5.9)	0	0.194	1 (5.6)	3 (2.9)	0.569

Data are shown as mean ± SD. SET, single embryo transfer; DET, double embryo transfer; HDP, hypertensive disorders of pregnancy; GDM, gestational diabetes mellitus.

## Discussion

This study is clinically relevant as it compared pregnancy outcomes of DCDA twin pregnancies conceived through two distinct MAR strategies: SET and DET. Although DCDA twins are generally regarded as the lowest-risk form of twinning, our data showed that SET-derived DCDA twins had a significantly higher incidence of complete miscarriage and a markedly lower rate of twin live birth than DET-derived DCDA twins. Multivariable logistic regression analysis further demonstrated that the associations between SET-derived DCDA twin pregnancies and poorer early outcomes remained directionally consistent after adjustment for key clinical and embryological factors. These findings indicate that DCDA twins should not be regarded as a uniform group, as their clinical risks differ depending on whether they originate from two separate embryos (dizygotic) or from the division of a single embryo (monozygotic). Thus, zygosity, rather than chorionicity alone, appears to be a key determinant of early pregnancy outcomes in DCDA twin pregnancies conceived through MAR.

MZT remains a critical embryological concern in MAR. Classical theory associates DCDA, MCDA, and MCMA twins with cleavage occurring within 3, 4–7, and 8–12 days post-fertilization, respectively (Benirschke and Kim, 1973; Hall, 2003; Redline, 2003). However, the increasing frequency of DCDA twins after blastocyst-stage SET cannot be explained solely by this timeline (Hviid *et al.*, 2018; Ikemoto *et al.*, 2018; Hattori *et al.*, 2019). Our previous multicenter retrospective analysis suggested that embryonic splitting leading to monozygotic twins likely occurs after the blastocyst stage, resulting from the separation of the ICM and TE (Kyono, 2013). Furthermore, a recent review on the cellular mechanisms underlying monochorionic twinning after SET proposed that gradual separation of the ICM during blastocoel expansion could lead to the formation of multiple ICM clusters within a single blastocyst (Jin *et al.*, 2024). Recent time-lapse analyses using human stem cell-based blastocyst models have shown that, during blastocoel expansion, the ICM cluster can elongate into a line or smeared configuration of cells that gradually thins in the center and subsequently reconsolidates into separate clusters (Luijkx *et al.*, 2024). For SET-derived DCDA twins, abnormal ‘figure-eight’ hatching and post-vitrification separation of the blastocyst have been proposed as potential mechanisms leading to dichorionic MZT (Yan *et al.*, 2015). Certain MAR manipulations may predispose embryos to post-transfer splitting. Prolonged *in-vitro* culture may weaken ICM adhesion, and AH might mechanically induce division as the embryo expands through a laser-induced opening (Alikani *et al.*, 1994; Jin *et al.*, 2024). Although some studies support these associations, others do not, and the causal relationship between AH and MZT remains unresolved (Busnelli *et al.*, 2019; Paul *et al.*, 2025). In our cohort, AH was universally performed in DET cycles and in 76.9% of SET cycles; however, the rate did not differ between SET-derived MCDA and DCDA twins. Differences in endometrial preparation between groups likely reflected evolving clinical practice, as HRC has been increasingly replaced by NC owing to reports of higher rates of placenta previa and large-for-gestational-age infants in HRC (Saito *et al.*, 2019). Vitrification–warming itself may also promote DCDA twinning: during blastocoel re-expansion, partial ICM–TE separation can occasionally generate two blastocyst-like structures within a single ZP (Shibuya and Kyono, 2012).

Although numerous studies have reported higher MZT rates after MAR, few have examined whether clinical outcomes differ between DCDA twin pregnancies following SET versus DET. Previous case series demonstrated that monozygotic DCDA twins can arise after single blastocyst transfer, but these reports were limited by very small sample sizes and did not compare them with dizygotic DCDA twins resulting from DET (Konno *et al.*, 2020; Li *et al.*, 2020). In our analysis, SET-derived DCDA twins experienced more frequent double fetal loss before heartbeat confirmation and lower twin live-birth rates than DET-derived DCDA twins. This disparity suggests that late-stage cleavage may result in an unequal allocation of the ICM or TE, giving rise to one or both embryos that are developmentally compromised. In animal models, particularly in sheep and cattle, artificial blastocyst bisection has been reported as an efficient method for generating monozygotic twins, with comparatively high developmental and birth success rates (Willadsen and Godke, 1984; Williams *et al.*, 1984). However, in non-human primates such as rhesus and cynomolgus monkeys, although embryo splitting at the early cleavage stage and subsequent culture and implantation have been achieved, no study has yet reported successful delivery of live offspring (Chan *et al.*, 2000; Mitalipov *et al.*, 2002). Single-cell RNA-sequencing studies have shown that in human embryos, epiblast (EPI), and primitive endoderm (PrE) lineages emerge within the ICM between Days 5 and 7 post-fertilization and subsequently undergo positional sorting (Yan *et al.*, 2013; Petropoulos *et al.*, 2016; Chousal *et al.*, 2024). Because the ICM remains highly plastic during this period, embryonic splitting at this stage could lead to imbalanced allocation of EPI and PrE precursors, which may reduce developmental competence and result in embryonic arrest. Accumulating evidence suggests that monozygotic DCDA twins after SET often arise from division of the ICM, rather than from symmetric cleavage at earlier developmental stages (Kyono, 2013; Sundaram *et al.*, 2018; Jin *et al.*, 2024). Such late-stage splitting may predispose embryos to quantitative and qualitative cellular imbalances, including reduced total cell number or unequal allocation of EPI and PrE lineages within the ICM. These alterations could compromise implantation stability and early placentation, thereby increasing the risk of complete miscarriage even in dichorionic twin gestations (Yan *et al.*, 2013; Petropoulos *et al.*, 2016; Litwinska *et al.*, 2020). Notably, the persistence of an increased complete miscarriage risk in SET-derived DCDA twins even after fetal heartbeat confirmation suggests that early developmental vulnerability may extend beyond initial embryonic viability. Such persistence of risk may reflect underlying developmental or placental insufficiency affecting both embryos, whereby the demise of one embryo could trigger secondary loss of the co-twin through local inflammatory or uterine contractile mechanisms (Ward *et al.*, 2018; Gurney *et al.*, 2022). Collectively, these observations indicate that DCDA twin pregnancies are biologically heterogeneous, with early pregnancy risk influenced by factors beyond chorionicity alone. Conversely, the relatively high vanishing-twin rate (≥30%) in DET-derived DCDA twins may reflect the poorer quality of one transferred embryo or MZT arising from a single embryo. From a clinical perspective, the present findings do not challenge the established role of SET as the preferred strategy for minimizing multiple pregnancies. Instead, they highlight the need to recognize SET-derived monozygotic DCDA twin pregnancies as a distinct subgroup with increased vulnerability during early gestation. When DCDA twin pregnancies are identified after SET, enhanced early counseling and closer first-trimester surveillance may be warranted, given the increased risk of complete miscarriage observed in this group. Early determination of chorionicity and careful monitoring of embryonic viability may assist in appropriate prognostic counseling. Moreover, the persistence of pregnancy loss risk even after fetal heartbeat confirmation raises the question of whether early pregnancy management, including luteal phase support, could benefit such cases; however, further prospective studies are required to determine whether such approaches can improve outcomes in SET-derived DCDA twin gestations.

Although DCDA twin pregnancies are traditionally considered to carry lower risks than MCDA twins, our analysis of monozygotic twin pregnancies derived from SET revealed a contrasting pattern. Specifically, monozygotic DCDA twin pregnancies showed lower rates of confirmation of two fetal heartbeats and twin live birth and a higher rate of complete miscarriage than monozygotic MCDA twin pregnancies. This finding suggests that chorionicity alone may be insufficient to fully explain early pregnancy outcomes in monozygotic twin gestations. One possible explanation is that monozygotic DCDA twins represent a biologically distinct subgroup in which embryo splitting occurs at a potentially unstable developmental stage, leading to compromised embryonic or placental development. In contrast, monozygotic MCDA twins may arise from splitting events that allow a more balanced allocation of embryonic and extraembryonic lineages.

Previous reviews comparing spontaneous and MAR-derived DCDA twins have reported increased placental and neonatal complications in MAR pregnancies compared with those conceived naturally (Qin *et al.*, 2016; Chen *et al.*, 2024). In our study, placental abnormalities and birth weight did not differ significantly between SET- and DET-derived DCDA twins. Thus, while early gestational outcomes diverged, perinatal outcomes among continuing pregnancies were largely equivalent.

These observations suggest that SET-derived monozygotic DCDA twins may possess intrinsic developmental fragility despite sharing the same chorionicity as dizygotic DCDA twins. Early determination of chorionicity and vigilant surveillance during the first trimester are therefore crucial. Vaginal progesterone supplementation in twin pregnancies, including those conceived spontaneously, has been reported to reduce the risk of early preterm birth and to decrease neonatal morbidity and mortality in women with a short cervix on ultrasound (Conde-Agudelo *et al.*, 2023). These findings raise the question of whether more intensive luteal support in MAR might help prevent early pregnancy loss, particularly in SET-derived DCDA twin gestations. The comparable perinatal outcomes among ongoing pregnancies provide reassuring evidence for clinical management once viability is established. The principal strength of this study lies in its single-center design, minimizing inter-operator variation in ultrasonography, fertilization, AH, and vitrification techniques. Limitations include the relatively small number of MZT cases and the absence of genetic confirmation of zygosity. Monozygosity was inferred from early ultrasonography; thus, same-sex DCDA twins of dizygotic origin could not be fully excluded. Nevertheless, given the exclusion of discordant-sex twins and the extreme rarity of spontaneous dizygotic twinning after SET, most SET-derived DCDA twins in this cohort can reasonably be regarded as monozygotic. It is also possible that a small proportion of same-sex DCDA twin pregnancies in the DET group were monozygotic. Such misclassification could bias the observed differences in pregnancy outcomes in either direction. Therefore, future studies incorporating genetic confirmation of zygosity are required to validate these findings. In addition, the incidence of multiple pregnancies following SET observed in the present study (0.5%) was lower than the MZT rates reported in some previous studies (0.97–2.35%) (Vitthala *et al.*, 2009; Busnelli *et al.*, 2019). The reasons for this difference are not fully understood but may be related to several factors, including differences in study design, patient selection, and standardized clinical protocols at a single center, such as culture media, the type or timing of AH, and embryo quality.

In conclusion, DCDA twin pregnancies following SET showed a significantly higher incidence of complete miscarriage and a lower rate of twin live birth compared with those following DET. These findings indicate that even within the same chorionicity category, pregnancy outcomes differ according to embryological origin. While SET remains the preferred strategy to prevent multiple pregnancies and achieve singleton live birth, further investigation is required into the prognosis of SET-derived DCDA twins. Future studies should aim to identify blastocyst-stage embryos at greater risk of post-transfer splitting, such as those exhibiting atypical hatching or morphological irregularities, and to establish pre-transfer evaluation criteria to mitigate MZT. Integrating early ultrasonographic assessment with postnatal genetic confirmation of zygosity will be essential for elucidating the mechanisms of twin formation and optimizing perinatal outcomes.

## Data Availability

The data underlying this article will be shared on reasonable request to the corresponding author.
